# Three-Dimensional Probabilistic Maps of Mesial Temporal Lobe Structures in Children and Adolescents’ Brains

**DOI:** 10.3389/fnana.2018.00098

**Published:** 2018-11-15

**Authors:** Antoine Bouyeure, David Germanaud, Dhaif Bekha, Victor Delattre, Julien Lefèvre, Charlotte Pinabiaux, Jean-Francois Mangin, Denis Rivière, Clara Fischer, Catherine Chiron, Lucie Hertz-Pannier, Marion Noulhiane

**Affiliations:** ^1^INSERM, CEA, Université Paris Descartes, Sorbonne Paris Cité, Neurospin, UNIACT, UMR1129, Gif-sur-Yvette, France; ^2^Université Paris Diderot, Sorbonne Paris Cité, AP-HP, Hôpital Robert-Debré, DHU Protect, Service de Neurologie Pédiatrique et des Maladies Métaboliques, Paris, France; ^3^CNRS, ENSAM, LSIS UMR 7296, Aix Marseille University, Toulon University, Toulon, France; ^4^Université Paris Ouest Nanterre La Défense, Laboratoire CHArt (EA 4004), Nanterre, France; ^5^CEA, University Paris Saclay, NeuroSpin, UNATI, Gif-sur-Yvette, France

**Keywords:** medial temporal lobe, probabilistic maps, development, hippocampus, parahippocampal gyrus

## Abstract

The hippocampus and the adjacent perirhinal, entorhinal, temporopolar, and parahippocampal cortices are interconnected in a hierarchical MTL system crucial for memory processes. A probabilistic description of the anatomical location and spatial variability of MTL cortices in the child and adolescent brain would help to assess structure-function relationships. The rhinal sulcus (RS) and the collateral sulcus (CS) that border MTL cortices and influence their morphology have never been described in these populations. In this study, we identified the aforementioned structures on magnetic resonance images of 38 healthy subjects aged 7–17 years old. Relative to sulcal morphometry in the MTL, we showed RS-CS conformation is an additional factor of variability in the MTL that is not explained by other variables such as age, sex and brain volume; with an innovative method using permutation testing of the extrema of structures of interest, we showed that RS-SC conformation was not associated with differences of location of MTL sulci. Relative to probabilistic maps, we offered for the first time a systematic mapping of MTL structures in children and adolescent, mapping all the structures of the MTL system while taking sulcal morphology into account. Our results, with the probabilistic maps described here being freely available for download, will help to understand the anatomy of this region and help functional and clinical studies to accurately test structure-function hypotheses in the MTL during development.

**Free access to MTL pediatric atlas:** http://neurovault.org/collections/2381/.

## Introduction

The medial temporal lobe (MTL) plays a pivotal role in memory and learning. During childhood and adolescence, memory abilities undergo a continuous development in relation to the anatomical maturation of MTL structures. These structures are the hippocampus (HC), divided along its rostrocaudal axis between the hippocampal head (HH), body (HB), and tail (HT), and the adjacent cortical areas of the parahippocampal gyrus (i.e., temporopolar, perirhinal, entorhinal, and parahippocampal cortices) to which the HC is reciprocally connected (Squire et al., 2004; Gogtay et al., 2006; Poppenk and Moscovitch, 2011; Poppenk et al., 2013; Strange et al., 2014). Together, HC subparts and adjacent cortices form a hierarchically organized system with both integrated functioning and structure-related specificities. Our understanding of the functional maturation of MTL structures during childhood and adolescence is, however, limited (e.g., DeMaster and Ghetti, 2013; DeMaster et al., 2013; Paz-Alonso et al., 2013; Pinabiaux et al., 2013; Riggins et al., 2016) compared to what is known in adults.

Taking into account the anatomical variability of MTL structures is a prerequisite to the study of their functional maturation during early years of life. Current anatomical and functional investigations of MTL in children and adolescents are constrained by the use of adult-based maps and landmarks that have not, or only partially, been adapted or confirmed in younger populations yet (e.g., DeMaster et al., 2013, 2014). Meanwhile, the complex sulcal pattern of the MTL defines a framework of anatomical landmarks to be used for locating the adjacent cortices (Insausti et al., 1998; Huntgeburth and Petrides, 2012; Augustinack et al., 2013a; Kivisaari et al., 2013). In fact, the rhinal sulcus (RS) and the collateral sulcus (CS) display various morphological conformations (Ono et al., 1990; Kim et al., 2008; Feczko et al., 2009; Huntgeburth and Petrides, 2012; Cikla et al., 2016). One of the most visible sulcal variation in the MTL is that the RS and CS can be either connected or separated (at a level slightly caudal to the caudal tip of the entorhinal cortex), which may impact the boundary localization, and the volume, surface area and cortical thickness of the adjacent cortices (Pruessner et al., 2002; Feczko et al., 2009). Still, these relations are poorly understood in adults and remain unexplored in children and adolescents. While in adults, probabilistic maps have already been proposed for the HC subfields, the perirhinal cortex and the entorhinal cortex (Amunts et al., 2005; Fischl et al., 2009; Augustinack et al., 2013a; Iglesias et al., 2015; Yushkevich et al., 2015), and for sulcal morphological variants (RS and CS proper) (Huntgeburth and Petrides, 2016), probabilistic description of the anatomical variability of all MTL structures (hippocampus, adjacent cortices, and sulci) in a group of children and adolescents has not been made. Such an atlas would be useful in several regards. First, using an adult atlas in children is limited by anatomical variation induced by development. The hippocampus is known to have a protracted structural maturation until adulthood that affects distinctly its head (anterior part) and tail (posterior part) (e.g., Gogtay et al., 2006), while the structural maturation of MTL cortices is almost unknown (see Hu et al., 2013). Second, mapping the anatomical variability of MTL sulci is relevant to understand the anatomical variability of the cortices they border. For example, the location of a functional region such as the parahippocampal place area is closely related to the morphology of the Collateral Sulcus (Huntgeburth and Petrides, 2016; Weiner et al., 2018). Third, an atlas of all MTL structures using a unified set of segmentation rules has never been made available. Therefore, a pediatric of all MTL structures, thereby taking into account age-related anatomical variability, would be useful to test structure-function relationships during development.

Here, we provide a detailed structural description of the MTL region in children and adolescents aged 7 to 17 years old, following automatic segmentation with the BrainVisa pipeline and according to a set of unified MTL manual segmentation rules for specific cortices that have been extrapolated from adult anatomical histological correlates as proposed in Hu et al. (2013) and Pinabiaux et al. (2013). For this purpose, we followed several steps, investigating successive hypotheses: (1) we confronted the RS-CS conformation observed in children and adolescents to that reported in adults to determine developmental characteristics of sulcation in the MTL; (2) we investigated whether the sulcal conformation in children and adolescents was related to age, sex and brain size; (3) we searched for an effect of the RS-CS conformation on morphometrical features of these sulci; (4) we searched for a difference in sulci location between the two RS-CS conformations at the group level, in the normalized space; (5) we generated probabilistic maps of each structure (RS, CS, cortices of the parahippocampal gyrus and HC subparts), *i.e.*, maps representing the variability of the location of anatomical structures after normalization in the MNI space, based on relative occurrence at the voxel level across subjects (Amunts et al., 2005; Eickhoff et al., 2005) to obtain an atlas of MTL structures during childhood. These maps are freely available for visualization and download on NeuroVault^1^.

## Materials and Methods

### Population

We studied 38 healthy subjects aged from 7 to 17 years (*M* = 11.71, *SD* = 3.03). There were 19 girls (*M* = 12.93, *SD* = 3.13) and 19 boys (*M* = 10.15, *SD* = 2.15). All subjects were right-handed and none had any history of medical condition. The study was approved by an appropriate research ethics board (CPP number 11-008). All subjects agreed to participate and their parents gave their informed consent to the study.

### Neuroimaging Data

MRI data were acquired on a 3T scanner device (Tim Trio, Siemens Medical Systems, Erlangen, Germany) with a 3D MPRAGE T1-weighted high-resolution sequence (TR: 2300 ms; TE: 2.98 ms; FOV: 256 mm; 64^∗^64 matrix; 160 sagittal slices, 1 mm3 isotropic) and analyzed with BrainVisa (v.4.4.0)^2^ including the Anatomist visualization module and the Morphologist segmentation pipeline (Rivière et al., 2009).

### Sulcal Analysis

#### Extraction, Correction, and Classification Into Morphological Sulcal Types

The Morphologist pipeline performs the automatic segmentation of both hemispheres, gray matter (GM) and white matter (WM) masks, then computes the inner cortical surface (GM-WM interface) and the outer cortical surface (pial surface) meshes, and finally sulcal proxies that are casts of the sulci with both a surfacic (mesh) and volumetric (voxel cluster) representation. The last step of the pipeline consists in anatomical labeling of the sulci through a localization-based probabilistic method named Statistical Probabilistic Anatomy Map (SPAM) (Perrot et al., 2011).

**FIGURE 1:**
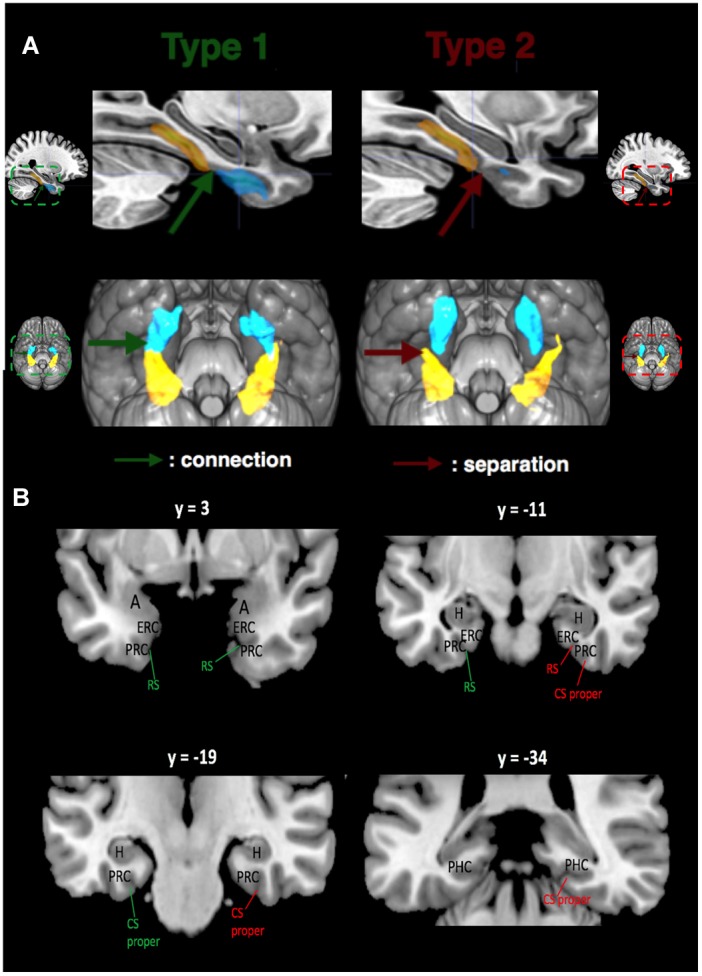
Relations between CS proper (in yellow) and RS (in blue) for both sulcal Types shown with mean sulcal maps. **(A)** Left: Mean Type 1 RS and CS proper are connected (shown by green arrow). Right: Mean Type 2 RS and CS proper are separated (shown by red arrow). Mean RS and CS proper (at the population level) for Types 1 and 2 are surimposed on the MNI152 template of MRIcro. **(B)** Location of MTL structures. MNI coordinates are provided for the y-axis. The same color code is used: green indicates connection between RS and CS proper, while red indicates separation. Abbreviations: A, amygdala; H, hippocampus; ERC, entorhinal cortex; PRC, perirhinal cortex; PHC, parahippocampal cortex.

Automatical labeling of CS and RS was corrected manually when necessary in each hemisphere; the SPAM method often mislabels collateral branches or segments of the CS and RS, mistaking them, for instance, with parts of the neighboring occipito-temporal sulcus. Indeed, following nomenclature of MTL sulci, we distinguished between RS and CS rather than labeling the RS as the ‘rostral part’ of the CS (see Insausti et al., 1995; Pruessner et al., 2002; Huntgeburth and Petrides, 2012 for review). Both manual correction of CS-RS boundary and labeling relied on a reference atlas (Ono et al., 1990) and on a recent study (Huntgeburth and Petrides, 2012), based on which we further distinguished two components to the CS: the CS proper and the CS post. Basically, the RS is more rostral and medial, delineating the entorhinal cortex (ERC) and the rostral perirhinal cortex (PRC) (see Figure 1A). The CS proper is more caudal and lateral, delineating the PRC and the parahippocampal cortex (PHC), while the CS post starts caudally to the caudal bit of the CS proper, extending into the occipital lobe (Ono et al., 1990; Huntgeburth and Petrides, 2012; Kivisaari et al., 2013; Chau et al., 2014; Lehman et al., 2016). The caudal border of the uncus was used to delimit the caudal border of the RS. For the CS (taken as whole), the caudal border of the uncus and the position of the amygdala delineated its rostral border, while the most caudal tip of its main medial bank delineated the caudal border. The CS was then separated into rostral (CS proper) and caudal (CS post) segments. The caudal tip of the body of the HC, at the level of the splenium of the corpus callosum as seen in coronal view in the MNI coordinates system, was used as a transition landmark between CS proper and CS post (Huntgeburth and Petrides, 2012). This landmark was also used to delineate the caudal border of the PHC. Hence, the limit between CS proper and CS post (Huntgeburth and Petrides, 2012) is deemed to coincide with the transition from memory-allocated cortices in the parahippocampal gyrus to vision-related cortices in the lingual gyrus.

After extraction and correction, we categorized the MTL sulcal patterns into two types based on either the connection (Type 1) or the separation (Type 2) between RS and CS proper (Figure 1B). In the same fashion, CS post was labelized as Type 1 when it followed caudally a Type 1 CS proper, and Type 2 otherwise. Morphometric measurements (maximum depth of the sulcus, mean depth of the sulcus, and length of the sulcus) were performed for each sulcus in the native space with the dedicated BrainVisa tool.

#### Probabilistic Maps of MTL Sulci

Masks of the accurately labeled RS, CS proper and CS post in each hemisphere of each subject were extracted with BrainVisa’s module Anatomist The masks were then normalized in the MNI stereotaxic space with SPM12 using the ‘old normalize’ tool^3^. Each mask was smoothed with a 3 mm Gaussian kernel to increase the continuousness of the map. Mean volumes were computed over all subjects for each sulcus of interest, separately for each hemisphere. Therefore, in the obtained maps, the voxels intensities range from 0 (voxel absent in every individual mean map) to 1 (voxel present in every individual mean map). Finally, a threshold of 5% was applied to get rid of outlier voxels (*i.e.*, to keep voxels that were present in at least 5% of subjects corresponding to at least one subject over the whole group). These maps were superimposed on the MNI152 1 mm brain mask.

### Mesial Temporal Structures Analyses

#### Manual Segmentation of MTL Cortices and HC Subparts

**FIGURE 2:**
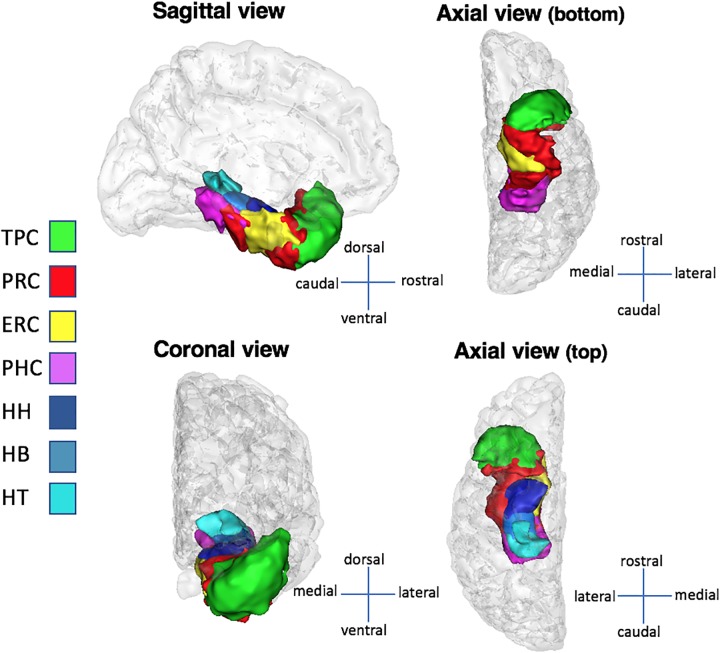
Medial temporal lobe structures in sagittal, coronal, and axial views. The structures presented here are computed over the whole population and converted to surfaces with BrainVisa’s AimsMesh function. A threshold of 5% (the same used volumes visualization) was applied on the surfaces to keep voxels present in at least 95% of subjects. Abbreviations: TPC, temporopolar cortex; ERC, entorhinal cortex; PRC, perirhinal cortex; PHC, parahippocampal cortex; HH, hippocampus head; HB, hippocampus body; HT, hippocampus tail.

Medial temporal lobe cortices (ERC, PRC, PHC, and TPC) and HC subparts (HH, HB, and HT) were manually delineated on both sides in each subject (Figure 2). Manual segmentation of MTL regions was done accordingly to the Insausti et al. (1998) protocol, except for the PHC that was segmented in reference to the protocol of Pruessner et al. (2002), as we did in some of our previous works (Noulhiane et al., 2006, 2007; de Vanssay-Maigne et al., 2011; Pinabiaux et al., 2013). The HC subparts were segmented according to criteria detailed in Kivisaari et al. (2013). Details are provided in Supplementary Table 1. Given our population of children and adolescents, we adjusted our criteria of HC segmentation according to hippocampal development (see Insausti et al., 2010). The translation of these criteria for the segmentation of MTL cortices and HC subparts in children and adolescents has already been proposed (e.g., Hu et al., 2013; Pinabiaux et al., 2013) and is deemed valid in the absence of conflicting data on anatomical histological correlation at that age.

#### Probabilistic Maps of MTL Cortices and HC Subparts

Probabilistic maps of MTL cortices and of HC subparts were generated with the same method than for sulcal probabilistic maps, at the exception of the smoothing step, which was not applied because cortical structures and HC subparts are larger than brain sulci, and therefore don’t need smoothing to reach important inter-subject overlap.

### Statistical Analyses

The following analyses were conducted, with a *p*-value of 0.05 as the threshold for significance for each analysis:

(1) We computed the proportion of Type 1 and Type 2 RS-CS proper conformations (which were observed in subject space for each sulci) and tested it against the proportions reported in adult literature with a one-proportion *z*-test (“proportion_*z*-test” function from python’s StatsModels package).
(2) We tested whether age, sex, brain size (volume and surface) and sulcal morphometric features, as measured in subject space, were correlated to RS-CS proper conformation, with *t*-tests (*t*-test_ind function Python’s SciPy package), corrected for multiple comparisons with FDR (“fdr correction” function from python’s statsmodels package).
(3) We assessed the effect of the RS-CS proper conformation on the MLT sulcal and cortical spatial locations in normalized space. To that purpose, we located the extrema along the mediolateral, rostrocaudal and dorsoventral axes of each sulcus or cortex probabilistic blob, *i.e.*, the volume defined by the non-null voxels of the corresponding statistical map (already thresholded at 5%). This can be seen as a bounding box for the blob of the structure. For each extremum, we then computed the difference between Type 1 and Type 2 RS-CS proper conformation groups and tested its significance using permutation test: we simulated the distribution of differences between each extremum for 1000 pairs of groups of the size of Type 1 and Type 2 groups but randomly generated by permutation. The difference observed between real Type 1 and Type 2 groups was considered significant if superior to the 95th centile of the simulated random distribution. To test the sensitivity of the 5% threshold applied to the statistical map defining the blobs, we repeated the analysis with a 25% threshold, *i.e.*, excluding voxels that are present in less than 25% of the subjects. This analysis was performed with MATLAB’s permutation function “randperm.”
(4) We searched for differences between sulcal variations in the location of the center of mass of each sulcus in normalized space. Namely, we computed the center of mass of each sulcus (via Python’s package NumPy), separately for Type 1 and Type 2. We used permutation testing to assess the differences in the location of the center of mass for each axis (x, y, and z) between Type 1 and Type 2 sulci. Then, we analyzed the variability of these center of mass in terms of direction. For each MTL sulcus, we projected individual center of mass in 3D space separately for Type 1 and Type 2, and fitted the cloud of points with an orthogonal distance regression line using singular value decomposition (via MATLAB’s function ‘svd’). We thus obtained a parametric equation *P* = p0 + t^∗^d where p0 is the average position of the vector, d the directions of the vector in 3D space, and t the parametrical value for each point. We then compared the direction parameters between Type 1 and Type 2, for each sulcus, using paired *t*-test. This analysis is complementary to the previous one as it describes the variability of sulcal center of mass between Type 1 and Type 2 sulci regarding their principal direction.

## Results

### RS-CS Proper Conformation

Among the 76 analyzed hemispheres, 29 had a Type 1 pattern (38.15%) and 47 a Type 2 pattern (61.85%). The inter-hemispheric correlation between types was high (*r* = 0.71), meaning that both hemispheres of a same subject tended to share the same type. The differences in type proportions between the present study and previous studies were not significant using proportions *z*-tests: comparison between the present study with Ono et al. (1990): *p* = 0.16; with Novak et al. (2002): *p* = 0.86; with Kim et al. (2008): *p* = 0.40; with Huntgeburth and Petrides (2016): *p* = 0.89; with Chau et al. (2014): *p* = 0.75; and with Cikla et al. (2016): *p* = 0.58. More details about comparison with previous studies are to be found in the Discussion section of this paper.

### Age, Sex, and Brain Size Effects on RS-CS Proper Conformation

#### Age

No age effect was found on RS-CS proper conformation (left hemisphere: *t* = 1.14, *p* = 0.29, right hemisphere: *t* = 0.01, *p* = 0.98). Therefore, subjects were distributed into sulcal types regardless of age.

#### Sex

No sex effect was found on RS-CS proper conformation, both in the left (*t* = 0.01, *p* = 1) and right (*t* = 0.12, *p* = 0.74) hemispheres.

#### Brain Size

No hemispheric size effect was found on RS-CS proper conformation on both sides, neither on volumes (left hemisphere: *t* = 0.10, *p* = 0.74; right hemisphere: *t* = 0.06, *p* = 0.79) nor on surface [left: *F*(1,36) = 0.49, *p* = 0.48; right: *F*(1,36) = 0.14, *p* = 0.71].

### Morphometrical Measurements of the RS and CS According to RS-CS Proper Conformation

A summary of the morphometrical measurements of the RS and CS according to RS-CS proper conformation is presented in Table 1.

**Table 1:** Morphological measurements of the MTL sulci as measured in native space.

Sulcus name	Rhinal sulcus				Collateral sulcus proper				Collateral sulcus post			
Hemisphere	Left		Right		Left		Right		Left		Right	
Sulcal variant	Type 1	Type 2	Type 1	Type 2	Type 1	Type 2	Type 1	Type 2	Type 1	Type 2	Type 1	Type 2
Max depth in mm (SD)	15.27 (3.71)	13.60 (2.58)	16.17 (2.37)	13.64 (2.50)	16.48 (2.96)	15.16 (3.01)	16.93 (1.93)	16.45 (2.15)	16.15 (2.15)	16.17 (1.50)	16.40 (1.63)	16.36 (1.81)
Mean depth in mm (SD)	9.21 (1.54)	8 (1.52)	10.45 (1.24)	8.06 (1.26)	12.41 (2.18)	10.11 (2.18)	12.42 (2.16)	10.96 (2.17)	10.36 (1.10)	10.23 (0.95)	11.15 (1.43)	10.74 (1.07)

*The mean and maximum depth of each sulcus are given separately for Type 1 and Type 2. For each sulcus of each type of each hemisphere, the mean value is indicated (in mm) with its SD.*

In Type 1, the maximum depth of the RS was significantly larger than in Type 2, in the right hemisphere (*t* = -3.08, *p* < 0.01, corrected) but not in the left (*t* = -1.36, *p* = 0.36). For the mean depth of the RS, the right hemisphere was significant after correction (*t* = -5.76, *p* < 0.005, corrected), but not the left (*t* = -2.40, *p* = 0.02 before correction, *p* = 0.14 after correction).

For the CS proper, no significant differences between Types 1 and 2 were found for the maximum depth (left hemisphere: *t* = -1.34, *p* = 0.38; right hemisphere: *t* = -0.69, *p* = 0.54). However, the mean depth of the CS proper was significantly greater in Type 1 than in Type 2 in the right hemisphere (*t* = -2.8, *p* < 0.01, corrected). In the left hemisphere, no significant difference was observed after correction (*t* = -2.82, *p* = 0.008 before correction, *p* = 0.11 after correction).

No significant differences of the CS post were found between Types 1 and 2 for maximum and mean depth for both hemispheres.

### Effect of RS-CS Proper Conformation on Sulci Location: Permutation Tests

**FIGURE 3:**
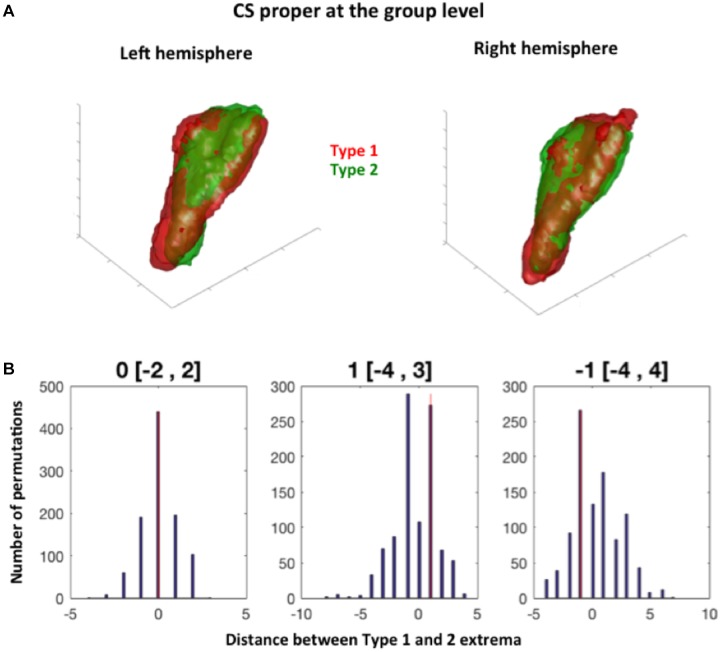
Permutation test procedure. **(A)** Two blobs are defined at the group level by merging the sulci of Type 1 and Type 2 subjects for a given sulcus (here illustrated with the collateral sulcus proper for both hemispheres). Here, the group-level Type 1 collateral sulcus proper is in red, and the group level Type 2 collateral sulcus in green. A bounding box illustrates the coordinates of these group sulci. **(B)** The extrema coordinates on the x, y, and z axis between the observed group sulci (shown in **A**) and 1000 group sulci generated randomly are compared.

To complete the analyses of the effect of sulcal conformation on sulcal features, we tested weather the observed conformations (i.e., Type 1 vs. Type 2) was related to different locations of Type 1 and Type 2 sulci of RS, CS and CS proper sulci in normalized space. The location of each mean sulcus (the sulcus computed over all subjects) was thus defined on each axis (i.e., x, y, and z axes) by pair of extrema, i.e., a minimum and a maximum location for each axis in the x, y, and z directions (Figure 3). Hence, the minima of a sulcus on the x-axis corresponds to the most medial point of the sulcus, while its maxima corresponds to the most lateral point; on the y-axis, the minima corresponds to the most rostral/anterior point, while the minima corresponds to its most caudal/posterior point; and for the *z*-axis, the maxima corresponds to the most dorsal point, while the minima corresponds to the most ventral point. The extrema were computed at two thresholds regarding the voxels included in the mean map of each sulcus: the 5% threshold, which was also used for visualization of the probabilistic maps (see section below), and a 25% threshold, to look at differences in location between mean sulci for each types that were present in at least 75% of subjects.

Regarding the minima of each sulcus along each axis, we found no significant differences between Type 1 and Type 2 RS, CS proper and CS post, at the 5 and 25% thresholds (Table 2). For the maxima (maximal coordinate of the blob along each axis), we found a significant difference between Type 1 and Type 2 RS for the x-axis in the right hemisphere at the 5 and 25% map thresholds. The observed difference in distance between Type 1 and Type 2 right RS maxima was of -5 mm at the 5% map threshold, a difference outside the 95% of the random distribution interval ranging from -2 to 3 mm. This means that in Type 1, the location of the right RS maxima is located significantly more laterally than in Type 2 RS at the 5% threshold. Similarly, the observed distance difference between Type 1 and Type 2 maxima was of 7 mm (outside the 95% of the random distribution interval ranging from -4 to 5 mm) at the 25% threshold, showing that in Type 1, the right RS maxima is located at a significantly less lateral level at the 25% threshold. The opposite sides of these differences suggested that, although Type 1 right RS extends more laterally than Type 2 right RS when looking at the mean map comprising nearly all subjects, the opposite is observed at the 25% threshold because the subject overlap of Type 1 right RS is less important laterally than for Type 2 right RS. No other significant differences were found for both hemispheres at both thresholds. Hence, overall, the location of the RS, CS proper and CS post was not greatly different between Type 1 and Type 2 patterns, with only one difference observed, regarding the location of RS maxima coordinates in the right hemisphere. These findings showed that the RS is deeper in the right hemisphere than in the left, as already shown by morphometrical measurements (see section “Morphometrical Measurements of the RS and CS According to RS-CS Proper Conformation”). We also tested differences in minima locations for each MTL cortex, finding accordingly no significant differences between the cortices bordered by Type 1 sulci, and those bordered by Type 2 sulci (data not shown).

**Table 2:** Distance between the minima and maxima of Type 1 and Type 2 for each sulcus, for two different thresholds.

Distance (in mm) between Type 1 and Type 2 minima.				Distance between Type 1 and Type 2 maxima.		
Left hemisphere				Left hemisphere		
RS	*x* axis	*y* axis	*z* axis	*x* axis	*y* axis	*z* axis
0.05 threshold	1 [-2, 3]	0 [-4, 14]	-2 [-2, 2]	0 [-3, 1]	0 [-6, 1]	0 [-4, 2]
0.25 threshold	-2 [-3, 2]	-3 [-8, 8]	-3 [-5, 2]	0 [-4, 3]	-1 [-7, 9]	-1 [-3, 2]
CS proper	*x* axis	*y* axis	*z* axis	*x* axis	*y* axis	*z* axis
0.05 threshold	2 [-2, 5]	1 [-3, 5]	8 [-8, 11]	0 [-4, 1]	1 [-6, 3]	0 [-2, 0]
0.25 threshold	-2 [-6, 4]	4 [-7, 8]	-1 [-6, 5]	-3 [-7, 5]	4 [-10, 8]	-2 [-5, 7]
CS post	*x* axis	*y* axis	*z* axis	*x* axis	*y* axis	*z* axis
0.05 threshold	14 [-14, 15]	10 [-10, 14]	7 [-7, 8]	0 [-7, 1]	0 [-4, 3]	0 [-10, 5]
0.25 threshold	3 [-6, 6]	0 [-10, 11]	0 [-5, 4]	18 [-13, 19]	1 [-26, 12]	7 [-5, 9]

**Right hemisphere**				**Right hemisphere**		
**RS**	***x* axis**	***y* axis**	***z* axis**	***x* axis**	***y* axis**	***z* axis**

0.05 threshold	1 [-3, 2]	-6 [-9, 4]	3 [-5, 4]	-5 [-2, 3]^∗^	-3 [-3, 6]	7 [-3, 8]
0.25 threshold	2 [-2, 3]	0 [-6, 9]	2 [-6, 3]	7 [-4, 5]^∗^	-3 [-5, 5]	-2 [-6, 5]
CS proper	*x* axis	*y* axis	*z* axis	*x* axis	*y* axis	*z* axis
0.05 threshold	-3 [-4, 2]	-1 [-3, 2]	-2 [-4, 3]	1 [0, 3]	4 [-3, 7]	-1 [-3, 2]
0.25 threshold	1 [-2, 3]	0 [-4, 3]	0 [-3, 5]	1 [-2, 2]	2 [-5, 3]	1 [-3, 3]
CS post	*x* axis	*y* axis	*z* axis	*x* axis	*y* axis	*z* axis
0.05 threshold	2 [-2, 6]	-1 [-9, 8]	1 [-4, 4]	-1 [-2, 3]	-1 [-1, 3]	0 [-8, 7]
0.25 threshold	1 [-2, 2]	2 [-5, 3]	1 [-3, 3]	1 [-10, 8]	-9 [-17, 12]	-1 [-5, 5]

*Permutation tests were used to compare the distance (in mm) between the minimum (i.e., minima) coordinates of the Rhinal Sulcus, Collateral Sulcus Proper, and Collateral Sulcus Posterior for the x, y, and z axis (i.e., the ‘x,’ ‘y,’ and ‘z’ lines of the table). The value outside of the brackets is the observed distance between the minima and maxima of Type 1 and Type 2 sulci, for each sulcus, separately for each axis. The values inside the brackets are the confidence interval of distance values, as computed with 1000 permutation tests. If the observed distance is included in the confidence interval, then it is not significantly different between Type 1 and Type 2 sulci. ^∗^Significant at *p* < 0.05. Two different thresholds were used: 0.05 (i.e., voxels present in at least 5% of the population) and 0.25 (voxels present in at least 25% of the population).*

We completed our analyses of sulcal extrema by testing differences in center of mass between Type 1 and Type 2 sulci. For each sulcal structure, we computed the individual center of mass of each sulcus and tested differences of the center of mass’ coordinates separately for each axis (x, y, and z) between Type 1 and Type 2 sulci. We found no significant differences between the center of mass of Type and Type 2 variants (Supplementary Table 2). Then, we tested the variability of sulcal center of mass in terms of direction. For each sulcus, we projected individual center of mass in 3D space separately for Type 1 and Type 2, and fitted the cloud of points with an orthogonal distance regression line using singular value decomposition. We compared the direction parameters of the line between Type 1 and Type 2 using paired *t*-test, and found no significant differences (Supplementary Table 3). Taken together, these results show no clear difference in location or direction between Type 1 and Type 2 sulcal variants. Hence, the effect of sulcal conformation seems limited, at least when analyzed in normalized space.

### Probabilistic Maps of the MTL Sulci and Cortices

#### Probabilistic Maps of MTL Sulci

**FIGURE 4:**
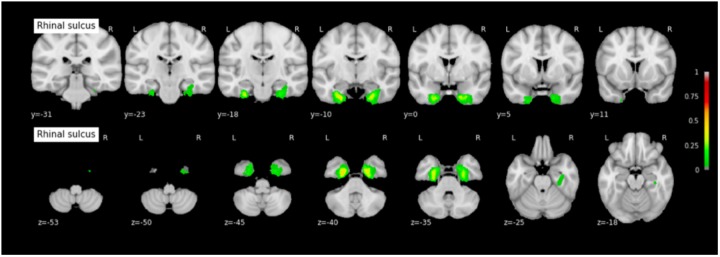
Probabilistic map of the rhinal sulcus (RS). The maps are normalized into the MNI stereotaxic space. Coordinates are indicated on each slice. Color bars indicate the probability of presence of the structure, ranging from 0 (voxel absent in subjects) to 1 (voxel present in subjects). A 0.05 threshold was used for visualization purposes in order to get rid of potential outlier voxels and to limit the extension of the maps caused by the 3 mm Gaussian kernel smoothing.

**FIGURE 5:**
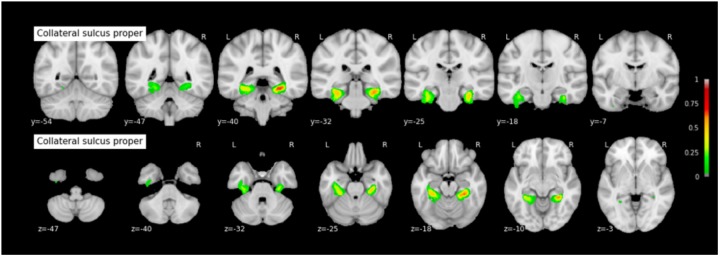
Probabilistic map of the collateral sulcus proper (CS proper). The maps are normalized into the MNI stereotaxic space. Coordinates are indicated on each slice.

**FIGURE 6:**
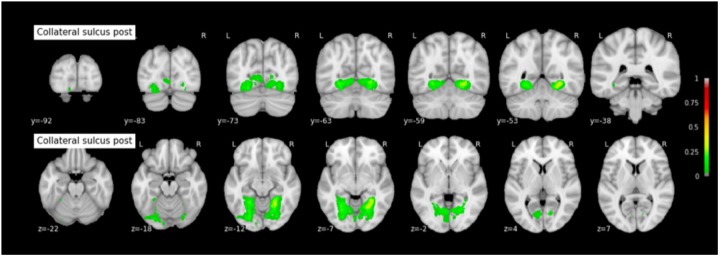
Probabilistic map of the collateral sulcus post (CS post). The maps are normalized into the MNI stereotaxic space. Coordinates are indicated on each slice.

Because permutation tests conducted in section “Effect of RS-CS Proper Conformation on Sulci Location: Permutation Tests” revealed few significant anatomical differences between Type 1 and Type 2 RS, CS proper and CS post, the probabilistic maps described here were generated by grouping together all subjects regardless of their sulcal type. The coordinates of each sulcus on the rostrocaudal axis (x axis) and on the dorsoventral axis (x-coordinates) are presented in Table 3. The probabilistic maps thresholded at 5% are shown in Figures 4–6.

**Table 3:** Coordinates of the MTL sulci in the MNI space.

MTL structure	RS	CS proper	CS post	ERC	PRC	TPC	PHC	HH	HB	HT
*y* coordinates	11; -31	-7; -54	-38; -92	6; -31	12; -38	29; 0	-22; -49	-4; -27	-19; -37	-29; -45
*z* coordinates	-18; -53	-3; -47	7; -22	-19; -49	-11; -54	-9; -53	-2; -35	-8; -33	-1; -26	9; -13

*The minimum and maximum coordinates of each sulcus are indicated for the rostrocaudal and dorsoventral axes.*

#### Probabilistic Maps of the MTL Structures

**FIGURE 7:**
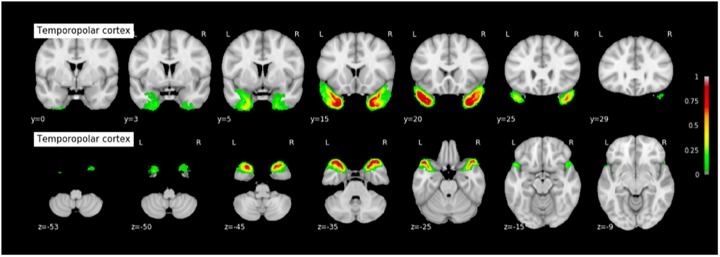
Probabilistic map of the temporopolar cortex (TPC). The maps are normalized into the MNI stereotaxic space. Coordinates are indicated on each slice.

**FIGURE 8:**
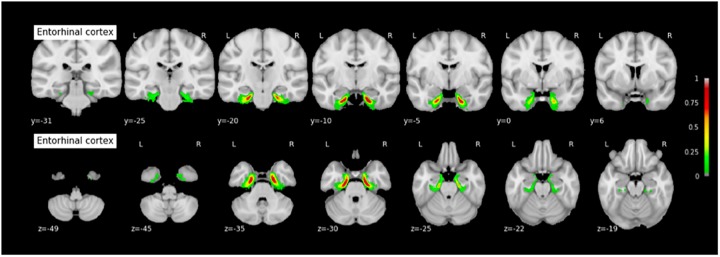
Probabilistic map of the entorhinal cortex (ERC). The maps are normalized into the MNI stereotaxic space. Coordinates are indicated on each slice.

**FIGURE 9:**
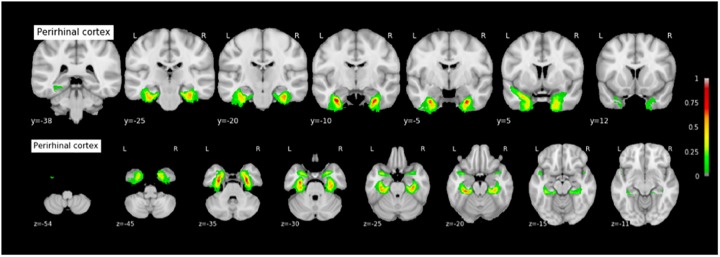
Probabilistic map of the perirhinal cortex (PRC). The maps are normalized into the MNI stereotaxic space. Coordinates are indicated on each slice.

**FIGURE 10:**
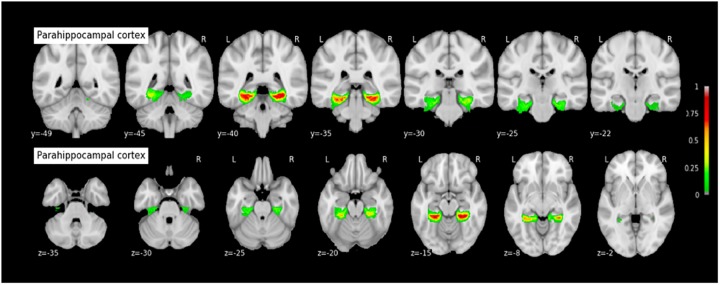
Probabilistic map of the parahippocampal cortex (PHC). The maps are normalized into the MNI stereotaxic space. Coordinates are indicated on each slice.

**FIGURE 11:**
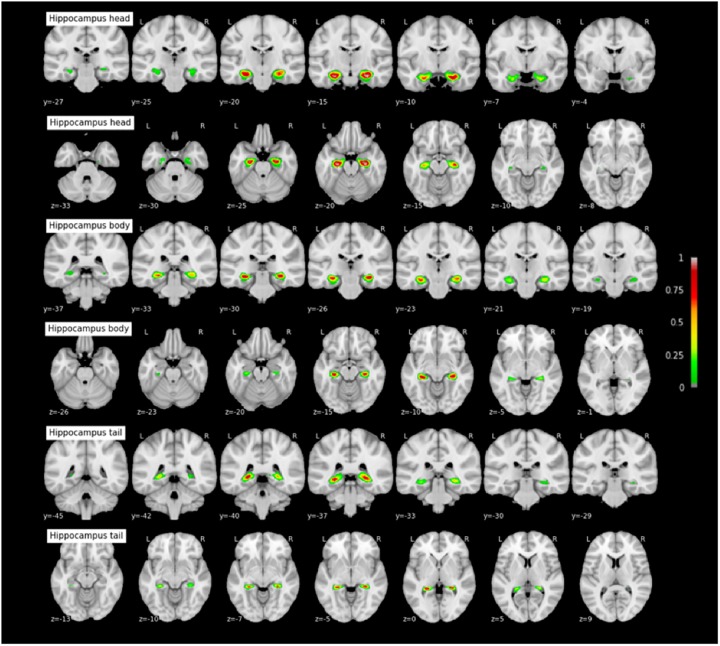
Probabilistic maps of the hippocampal subparts. The maps are normalized into the MNI stereotaxic space. Coordinates are indicated on each slice.

The coordinates of each MTL structures on the rostrocaudal axis (x axis) and on the dorsoventral axis (x-coordinates) are presented in Table 3. The probabilistic maps thresholded at 5% are shown are shown in Figures 7–11.

## Discussion

We characterized the spatial variability of MTL sulci and cortices in a child and adolescent population using probabilistic maps that constitute a MTL atlas. Such an atlas provides the expected location of each structure in a 3-dimensional stereotaxic space according to validated segmentation rules.

### MTL Sulcal Conformation in Children and Adolescents

#### Comparison With Adult Data

To date, no study had described the morphological characteristics of the RS, CS proper and CS post in a children and adolescents population. As explained in section “RS-CS Proper Conformation,” the description provided here uses the same morphotypes (connection vs. separation of the RS and CS proper) as previous studies. The Type 1 vs. Type 2 found in our study are comparable to previous adult studies (Table 4), as shown by proportion *z*-test (section “RS-CS Proper Conformation”).

**Table 4:** Sulcal morphology in children and teenagers compared with adults.

Study	Type 1 (%)	Type 2 (%)	Number of subjects
Present study (Bouyeure et al.)	38.15	61.85	38
Ono et al., 1990	28	72	25
Novak et al., 2002	36	64	50
Kim et al., 2008	45	55	51
Huntgeburth and Petrides, 2012	36.25	63.75	40
Chau et al., 2014	50	50	30 Formalin-fixed hemispheres
Cikla et al., 2016	42.9	57.1	35

*The proportions of three major morphological markers are compared between the present study and previous studies conducted in adults.*

Overall, the absence of differences between our results and previous adult studies regarding proportions of Types 1 and 2 suggests that one of the main effects of sulcation in this region, i.e., determining sulcal conformation, was achieved way before the age range studied here (7 to 17 years old). Interestingly, imaging studies of early development showed that most sulci appear during the 3rd gestational semester (Dubois et al., 2008); since CS and RS are visible as early as the 23th to the 25th gestational weeks on (Kier et al., 1997; Garel et al., 2003; Bajic et al., 2010), their sulcation may be achieved particularly early; here, we confirm that the morphogenesis of MTL sulcal conformations does not evolve after middle childhood.

#### Effect of Age, Sex, and Brain Volume on Morphometric Features

We found no effect of hemispheric volume on sulcal conformation. This may be surprising since Type 1 has been associated with deeper sulci (Feczko et al., 2009), a finding that we replicated in our population (see section “Morphometrical Measurements of the RS and CS According to RS-CS Proper Conformation”). Since larger brains are twistier because of the ramifications to accommodate the allometric increase of cortical surface (Germanaud et al., 2012), one could have expected hemispheric volume and surface to be positively associated to sulcal confluence and deepening, and therefore to Type 1. A recent study on the heritability of sulcal pits (Le Guen et al., 2017), i.e., locally deepest points in cortical sulci with little inter-subject variability, found that the Rhinal and Collateral sulci were amongst the most heritable brain sulci. These findings together suggest that the morphological variability of the Rhinal and the Collateral sulci could primarily be explained by specific genetic factors, independently of age, sex and brain size. Combined with the absence of effect of age on the proportion of sulcal variants, these findings suggest that the local variability of the MTL sulci is likely explained by specific genetic factors with very early influence.

### Effect of Sulcal Conformation on the Variability of Sulcal Location

We did not observe any significant differences in location between Type 1 and Type 2 probabilistic maps, although both types are clearly distinguishable at the subject level, and some morphometric features, such as mean and maximum depth, show significant differences between these two types (as measured in the native space). One first explanation could be that the normalization process may have minimized variability due to sulcal conformation, blurring both the differences in morphometric features measured in the native space and the difference in relative spatial location. Conversely, it could rather relate to the remaining variability after normalization due to a poor alignment of sulci since we used a normalization process not optimized for that purpose. Overall, the normalization process remains a key or even limiting step when it comes to group analysis of sulcal morphology and surface location (Lancaster et al., 2010; Lerch et al., 2017), and some teams try to avoid it (Mangin et al., 2016). We selected a very classical group normalization process as a first attempt to measure the impact of RS-CS proper morphotype on MLT cortices localization to stay close to the routine functional MRI procedure. Further work is needed to investigate whether the two sulcal conformations can be distinguished in terms of spatial location or have a significant impact on MLT cortices location by using other normalization procedures meant to preserve sulcal characteristics, such as a DARTEL normalization, or HIP-HOP model-driven harmonic parametrization of the cortical surface: (Auzias et al., 2013; Mangin et al., 2016). Machine learning could also be used to investigate whether sulcal conformation can be predicted from extrema location and other morphometric features. Nevertheless, it remains likely that the sulcal variation described here (Type 1 vs. Type 2) is not associated with localization differences large enough to cause significant differences at the group level in the normalized space, whatever the normalization process. The methodology used in the present work innovatively investigates the effect of sulcal variants on the anatomical location of said variants, using rigorous permutation testing. This method could be used or expanded in further work interested in investigating the relationships between anatomical variants of a given structure and the anatomical location of these variants, or the anatomical location of a given structure between different groups (e.g., groups based on age or condition).

### Relevance of the Probabilistic Maps: A MTL Atlas in Children and Adolescents

Probabilistic maps of MTL cortices have been generated in adults for PRC, ERC, and HC (Amunts et al., 2005; Augustinack et al., 2013a,b; Yushkevich et al., 2015). Comparison of these maps (implemented in SPM8’s toolbox ‘Anatomy’; see Eickhoff et al., 2005; or in FreeSurfer; Augustinack et al., 2013a) with the maps presented here shows similar locations and similar patterns of spatial variability. However, in the absence of systematic statistical comparison in location with probabilistic maps designed in adults to confirm a clear absence of changes in location during development, what we provide here is first-hand data regarding the location of TPC and PHC (for which no probabilistic map were available), plus PRC, ERC, and HC subparts, in a children and adolescents population. These maps, that are freely available for download, could be used by the community to easily generate mean masks of said structures when necessary, e.g., to verify the location of activation peaks during task-fMRI study, or to be used as ROIs for resting-state or DTI studies. For the HC, we provide here maps for each of HC subparts using the tripartite head/body/tail division, allowing to test for specific hypotheses regarding the anatomical and functional specialization of the hippocampus on its long-axis during development (e.g., Riggins et al., 2016; Blankenship et al., 2017). This point is particularly important, as several studies have shown that the hippocampus undergoes a protracted maturational process on its long-axis until early adulthood (Gogtay et al., 2006), while the anterior and posterior parts of the hippocampus are involved into different memory processes (Poppenk et al., 2013; Strange et al., 2014), with specific maturational dynamics for each HC subpart (Ghetti and Bunge, 2012; Riggins et al., 2015, 2016; Blankenship et al., 2017). Moreover, the tripartite head/body/tail division used here allows to test more fine-grained hypotheses of HC specialization than the anterior/posterior division that is also used by some studies. Our study has several limitations. First, the wide age range (7–17 years old) of our sample and our sample size (38 subjects) limits us from performing analyses for distinct age groups because of the small sample size that such groups would have. Therefore, a more comprehensive database of structural scans in the MTL would be beneficial in the future to assess more precisely the anatomical variation of this region during development. In particular, the structural maturation of the MTL region during early childhood is poorly known (except regarding the hippocampus: for example see Ngo et al., 2017; Canada et al., 2018; Riggins et al., 2018, for recent findings), while the first years of life see stark improvements of MTL-based mnemonic competences. It might interesting to apply similar methods to the ones outlined here (probabilistic mapping of anatomical variability, morphometry) to study MTL maturation in young children. A second limitation is that the SPM preprocessing procedure is less optimal than other co-registration and normalization methods. Our measure of the impact of RS-CS proper morphotype on MLT cortices localization aimed at staying close to the routine functional MRI procedure since it is in this context that functional studies, for instance, use MTL sulci as anatomical landmarks to identify cortical regions where activation peaks are located (see Huntgeburth and Petrides, 2016; Weiner et al., 2018 for discussions regarding the relation between the CS proper and PHC function). However, because we kept the same protocol throughout the study, the probabilistic maps were preprocessed using SPM as well. A systematic comparison of available registration methods to devise the most suited procedure for generating a probabilistic atlas in a pediatric population (e.g., choice of registration method, creation of a group template) would be beneficial to future work. Finally, the reader should bear in mind that the present findings stem from a segmentation method of MTL structures based on scarce adult histological data extrapolated to children and adolescents, possibly limiting the accuracy of the present segmentations. This limitation could be overcome if histological data of MTL structures in children and adolescents become available for future work, or ultrahigh resolution MRI as a proxy. Nevertheless, the present findings will be beneficial to studies interested in MTL structures, e.g., that use seed-based functional connectivity techniques that need a precise definition of anatomical regions of interest. Thus, this probabilistic 7–17 year-old MTL atlas should reduce potential errors or approximations in neuroimaging pediatric studies. This may apply for instance in temporal lobe epilepsy studies, in which the lesion and the epileptogenic focus frequently involve the parahippocampal gyrus with significantly more frequent Type 1 than Type 2 patterns (Kim et al., 2008). The MTL is also involved in several major neurodevelopmental conditions, such as autism (Bachevalier, 1994) or schizophrenia (Falkai et al., 1988; Arnold et al., 1997; Penttilä et al., 2008).

## Conclusion

This work provides the first probabilistic atlas of MTL sulci and cortices in a population of children and adolescents, and shows that the variation in sulcal conformation (connection or separation between the RS and the CS proper) is not explained by age, gender or brain size. This finding suggests that the local variability of the structures is likely primarily explained by genetic factors, with very early influence and further stability over late childhood and adolescence. The probabilistic atlas have been made available online in open access (See text footnote^1^).

## Ethics Statement

This study was carried out in accordance with the recommendations of the French “Comité de Protection des Personnes” regarding research with minors, and of the “Agence Nationale du Médicament et des Produits de Santé,” with written informed consent from all subjects and of the persons legally in charge of the subjects. All subjects gave written informed consent in accordance with the Declaration of Helsinki. The protocol was approved by the Comité de Protection des Personnes (CPP number 11-008).

## Author Contributions

DG, MN, and CP acquired the neuroimaging data. AB performed sulcal segmentation and analyses with the assistance of CF and MN. MN and AB performed the cortical segmentation and analyses. DB, VD, DR, MN, and AB did the probabilistic maps. JL, DB, DG, AB, and MN did the statistical analyses. AB, DG, and MN wrote the article with the assistance of DR, J-FM, CC, and LH-P.

## Conflict of Interest Statement

The authors declare that the research was conducted in the absence of any commercial or financial relationships that could be construed as a potential conflict of interest.

## Footnotes

1 http://neurovault.org/collections/2381/

2 http://brainvisa.info

3 https://www.fil.ion.ucl.ac.uk/spm/software/spm12/
