# Supplementary material for: Three-Dimensional Probabilistic Maps of Mesial Temporal Lobe Structures in Children and Adolescents’ Brains
Source: Front Neuroanat. 2018 Nov 15;12:98. doi: 10.3389/fnana.2018.00098 (PMC6249374; doi:10.3389/fnana.2018.00098)
Supplement: Supplementary file 1 [file Table_1.DOCX]

**Supplementary Material**

**Supp. Mat.** **Table 1. Anatomical landmarks used for the segmentation of MTL structures**. The landmarks presented here are adapted from Kivisaari, Probst & Taylor (2013) review on manual segmentation protocols in the MTL. Specifically, we used the protocol from Insausti et al. (1998) for MTL cortices, except for the PHC that was segmented according to the Pruessner et al. (2002) protocol. The hippocampal subparts were segmented following criteria mentioned in Kivisaari, Probst & Taylor (2013) and reproduced above. Generally speaking, the segmentation protocol consisted of a volumetric analysis based on histological landmarks reported on T1-MRI, to propose relevant MRI landmarks. To delineate the MTL structures, the protocol implied to progress in a rostro-caudal direction along the MTL in a coronal plane (1mm section) while checking the delineation in the other planes (axial, sagittal, 3D).

**Supp. Mat.** **Table 2. Comparison of the center of mass between Type 1 and Type 2 sulci.** The coordinates of the center of mass of each sulci were computed at the individual level. Permutation tests were used to compare these coordinates separately for each axis (x, y, and z) between Type 1 and Type 2 sulci. The p-values are in brackets and were not corrected for multiple comparisons.

**Supp. Mat.** **Table 3. Direction parameters of the orthogonal distance regression line and comparison between sulcal variants.** The parameters of the orthogonal distance regression line fitting the cloud of center of mass of all sulci are provided on the x, y and z directions, for sulcal variant of each sulcus of each hemisphere. Paired t-test were used to compare the parameters between Type 1 and Type 2. The t-values are indicated with the p-values in brackets that were not corrected for multiple comparisons.

**Table 1.**

|  | **Rostral border** | **Medial border** | **Caudal border** | **Lateral border** |
| --- | --- | --- | --- | --- |
| **Temporoplar cortex** | Temporal pole. | Most medial aspect of the parahippocampal gyrus. | Depends on the anterior border of the PRC. | First appearance of the gyrus of Schwalbe, superolaterally;  occipitotemporal sulcus, inferolaterally. |
| **Perirhinal cortex** | 2mm anterior to the most anterior coronal slice containing gray matter in the limen insulae, which coincides with the beginning of the RS. | Shoulder of the medial bank of the CS. It applies to the most medial one (i.e., to the RS) if the RS and the CS are discontinuous. | 4mm posterior to the disappearance the intralimbic gyrus. | Shoulder of the lateral bank of the CS, midpoint of the fusiform  gyrus, or midpoint between the fundus and the shoulder of the lateral bank  of the CS, depending on CS depth. |
| **Entorhinal cortex** | 2mm posterior to the first anterior slice where the white matter of the limen insulae is visible. | Midpoint of the gyrus ambiens or, if not visible, shoulder of the superomedial bank of the parahippocampal gyrus. | 1mm posterior to the last slice containing the apex of the intralimbic gyrus. | Midpoint of the medial bank of the RS. |
| **Parahippocampal cortex** | First slice after the posterior border of the PRC. | Medial apex of the parahippocampal gyrus. | First posterior slice where the pulvinar is no longer visible. | Adjusted to the depth of the CS, as for the PRC. |
| **Hippocampal head** | Most anterior corner of the conical profile formed by the parahippocampal gyrus white matter and the alveus. | Medial apex of the parahippocampal gyrus. | Apex of the intralimbic gyrus. | Medial wall of the temporal horn of the lateral ventricle. |
| **Hippocampal body** | One slice posterior to the posterior apex of the intralimbic gyrus. | Medial apex of the parahippocampal gyrus. | One slice posterior to the first coronalslice where the crus of the fornix is separated from the wall of the lateral ventricle. | Temporal horn of the lateral ventricle. |
| **Hippocampal tail** | First slice posterior to the posterior limit of the hippocampal body. | Gray matter superolateral to an oblique line draw on from the inferolateral corner of the angular bundle along the white matter of the parahippocampal gyrus to the quadrigeminal cistern. | Coincides to the disappearance of the ovoid-shaped mass of gray matter of the hippocampus. | White matter of the ascending crus of the fornix and the temporal  horn of the lateral ventricle. |

**Table 2.**

| **Sulcus** | **Rhinal sulcus** | | **CS proper** | | **CS post** | |
| --- | --- | --- | --- | --- | --- | --- |
| **Side** | **Left** | **Right** | **Left** | **Right** | **Left** | **Right** |
| **X** | -0.08 (0.88) | -2.42 (0.03) | 0.86 (0.39) | -0.30 (0.78) | -1.29 (0.21) | -1.76 (0.07) |
| **Y** | 0.14 (0.91) | -1.01 (0.34) | 1.84 (0.08) | 1.45 (0.15) | 0.24 (0.75) | -0.70 (0.50) |
| **Z** | -1.66 (0.12) | 1.41 (0.19) | -1.12 (0.28) | -0.23 (0.85) | 2.44 (0.01) | 0.19 (0.89) |

**Table 3.**

| Sulcus | Sulcal variant | X direction parameter | Y direction parameter | Z direction parameter | T-value of paired t-test (p-value) |
| --- | --- | --- | --- | --- | --- |
| Left RS | Type 1 | -0.12 | 0.75 | -0.64 | -0.95 (0.44) |
| Left RS | Type 2 | -0.22 | 0.89 | -0.38 |  |
| Right RS | Type 1 | -0.68 | -0.53 | 0.49 | -1.73 (0.22) |
| Right RS | Type 2 | 0.56 | 0.82 | 0.71 |  |
| Left CS proper | Type 1 | 0.30 | 0.68 | -0.66 | 0.40 (0.72) |
| Left CS proper | Type 2 | -0.45 | -0.70 | 0.53 |  |
| Right CS proper | Type 1 | 0.24 | -0.61 | 0.75 | 0.20 (0.85) |
| Right CS proper | Type 2 | -0.21 | 0.73 | -0.64 |  |
| Left CS post | Type 1 | 0.17 | 0.97 | 0.14 | 1.47 (0.27) |
| Left CS post | Type 2 | -0.24 | -0.96 | 0.03 |  |
| Right CS post | Type 1 | 0.03 | 0.99 | 0.07 | 2.25 (0.15) |
| Right CS post | Type 2 | -0.27 | 0.95 | -0.10 |  |
